# The use of agrobiodiversity for plant improvement and the intellectual property paradigm: institutional fit and legal tools for mass selection, conventional and molecular plant breeding

**DOI:** 10.1186/s40504-014-0014-7

**Published:** 2014-06-27

**Authors:** Fulya Batur, Tom Dedeurwaerdere

**Affiliations:** Research Fellow, College Thomas More, 1, Place Montesquieu, 1348 Louvain-la-Neuve, Belgium; Director of the Biodiversity Governance Research Unit at the Centre for the Philosophy of Law of the Universite catholique de Louvain, College Thomas More, 1, Place Montesquieu, 1348 Louvain-la-Neuve, Belgium

## Abstract

Focused on the impact of stringent intellectual property mechanisms over the uses of plant agricultural biodiversity in crop improvement, the article delves into a systematic analysis of the relationship between institutional paradigms and their technological contexts of application, identified as mass selection, controlled hybridisation, molecular breeding tools and transgenics. While the strong property paradigm has proven effective in the context of major leaps forward in genetic engineering, it faces a systematic breakdown when extended to mass selection, where innovation often displays a collective nature. However, it also creates partial blockages in those innovation schemes rested between on-farm observation and genetic modification, i.e. conventional plant breeding and upstream molecular biology research tools. Neither overly strong intellectual property rights, nor the absence of well delineated protection have proven an optimal fit for these two intermediary socio-technological systems of cumulative incremental innovation. To address these challenges, the authors look at appropriate institutional alternatives which can create effective incentives for *in situ* agrobiodiversity conservation and the equitable distribution of technologies in plant improvement, using the flexibilities of the TRIPS Agreement, the liability rules set forth in patents or plant variety rights themselves (in the form of farmers’, breeders’ and research exceptions), and other *ad hoc* reward regimes.

## Introduction

Within the more general Darwinian framework pertaining to the survival of the fittest in the wild, agricultural practices have always exerted "an evolutionary pressure on plants" (Kingsbury 2009). This pressure was initially characterised by the selection of the most efficient plants by farmers in order to sow them the following year, and intensified with the dawn of plant breeding, which metamorphosed crop improvement into a knowledge-intensive, extremely productive and fast-evolving research-and-development focused industry. As the domestication of plants epitomised the dawn of agriculture, it took almost ten thousand years for grain production to reach the impressive one billion tonnes mark in 1960, while the second billion mark was hit merely forty years’ later in 2000 (Khush 2001). Such impressive development is not associated with industrialisation, but rather with crop genetic improvement (Anderson *et al.*^1988^), which today indubitably stands as a sphere where returns on research investment remain "well above the returns attainable from alternative uses of funds" (Byerlee 1996; Evenson 2001; Gardner 2003). Controlled plant improvement can contribute to the achievement of sustainable agriculture and the alleviation of poverty by ensuring yield gains and efficient resource management (Khush 2001) even though other social, cultural, economic, environmental and policy factors may influence such results (Evenson and Gollin 2003). Yet this success also depends to a great extent upon the effective and equitable use of both wild and improved agricultural biological diversity, which constitutes an indispensable input to the entire range of modern plant-breeding science, and will likely play a growingly important role to tackle the challenges of climate change and population growth. Studies show that, although most breeding programmes remain based on former market successes, with 83 per cent of active selection research being conducted on the basis of standardised and improved varieties (Swanson 1997), researchers and breeders continue to utilise, and in fact depend upon ‘wild germplasm’ in order to ensure the long-term sustainability of their programs. Around 6.5 per cent of their breeding gene pool is indeed continuously maintained with wild species and “landraces” (Swanson 1997), defined by the Food and Agriculture Organisation (FAO) as “domesticated plants adapted to the natural and cultural environment in which they live (or originated); usually possessing more diverse phenotypes and genotypes than what is commonly referred to as a “breed”. Moreover, public and private breeders alike rely on traditionally non-proprietary materials that have been, and still may be, accessed through the international agricultural research community under the umbrella of the Consultative Group for International Agricultural Research (CGIAR). For instance, it is estimated that 75 per cent of all maize sold by private companies in Latin America in 1996 contained germplasm derived from material developed by CIMMYT, the International Maize and Wheat Improvement Centre (Morris and Lopez-Pereira 1999).

**Figure 1:**
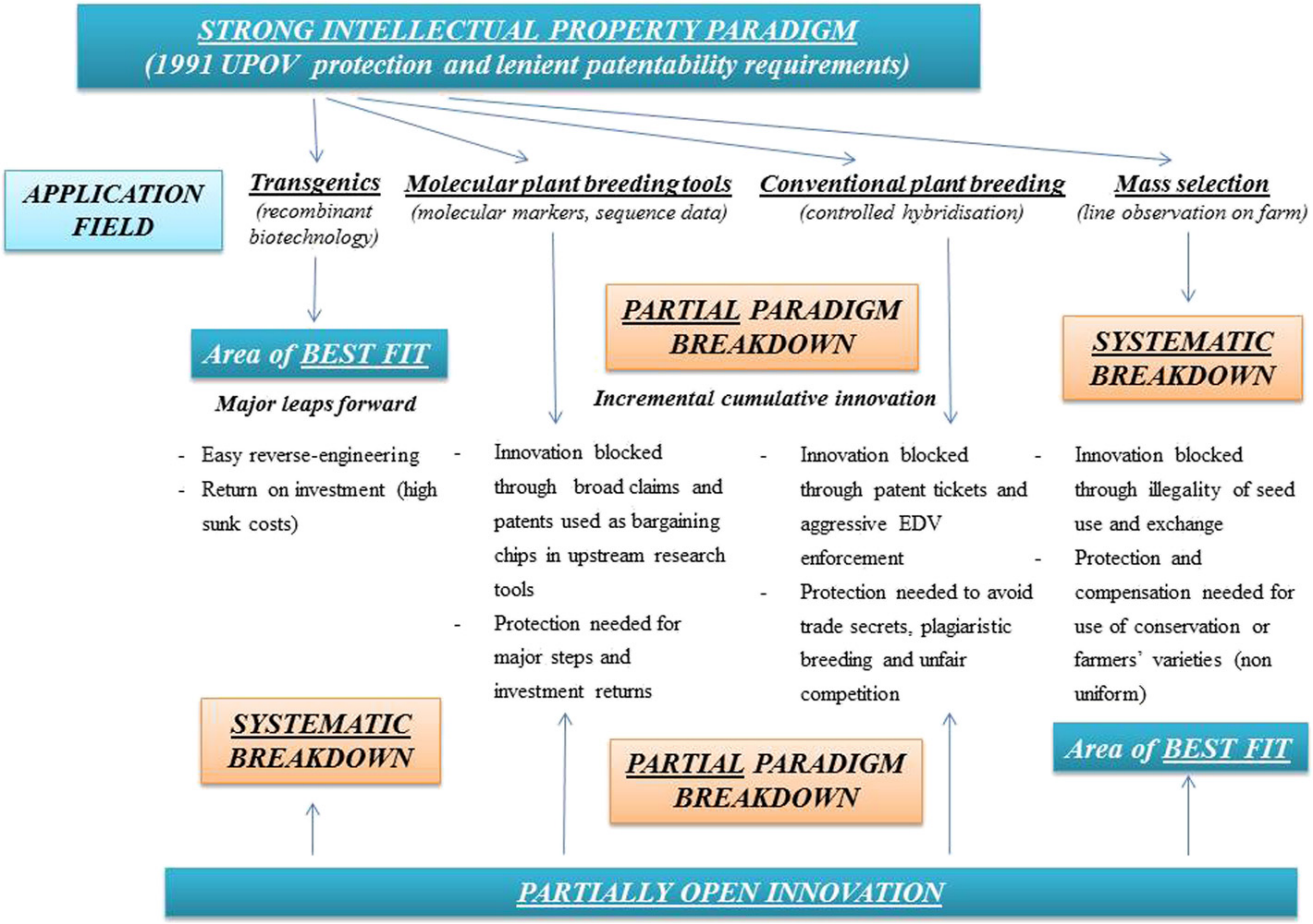
**Analysing paradigmatic fit: expected effects of the strong intellectual property paradigm and partially open innovation in the main fields of application of plant improvement (figure by authors).**

However, plant improvement faces a complex conundrum. Indeed, there is on the one hand an essential need to grant artificial lead time for research efforts through the recognition of intellectual property rights (IPR), in order to foster investment for the development of innovations that are easily reverse-engineered and costly to develop. While on the other hand, for follow-on innovators and cultivators, the prospects to use material from the protected pool of improved varieties have become increasingly conditional, with important detrimental effects, especially in a highly incremental innovation sphere such as plant improvement (Maskus and Reichman 2005). Follow-on uses of plant material or plant breeding techniques by farmers, breeders and scientists alike, have become remarkably complex on account of the growing number of IPR bestowed upon biological material or breeding techniques, especially following the adoption of the TRIPS Agreement, laying out the foundations of the strong intellectual property paradigm in its Article 27§3b. Furthermore, additional regulation on seed certification and market regulation, defining the conditions of use and distribution of both protected and non-protected improved varieties, has accentuated the shrinking range of manoeuvre left to those who operate or are pushed outside of such formal seed markets. The absence of apparent reward for local actors who conserve and at time upgrade the genetic resources upon which improved varieties are built on, further stresses the lack of regard for certain innovation systems. These detrimental aspects have led to growing criticisms of the dominant intellectual property paradigm in plant improvement, which have however not been very effective. Due to their piecemeal nature, critics highlighting genuine insufficiencies related to various areas, such as *in situ* agrobiodiversity, platform technologies and research tools or *ex-situ* pools of improved seed varieties have not yet produced a major shift in the paradigm; nor have the proposed alternative institutional tools been able to impose themselves as valid and viable institutional mechanisms. What is missing in such piecemeal approaches to the institutional effectiveness and/or defects of the protection of agrobiodiversity-related intangibles, in our view, is a systematic analysis of the relationship between institutional paradigms, such as intellectual property rights, and their context of application (Figure 1).

Our main hypothesis is that, in plant improvement, the opposition between the market based strong intellectual property paradigm, and the public-domain oriented fully-open angle as the main alternative paradigm, is too restrictive. We argue that the analysis of the context of application of intellectual property rights can provide better guidance for the future development of appropriate institutional alternatives to face the problems of *in situ* agrobiodiversity conservation and for a more equitable distribution of controlled hybridisation technologies and molecular biology research tools in plant improvement. We also argue that these institutional alternatives can be found within the flexibilities of either the TRIPS Agreement (allowing for effective *sui generis* protection of plant varieties, and loosely setting the contours of protection requirements), or within IPR tools themselves in the form of liability rules, such as compulsory licensing obligations, and also the farmers’, breeders’ and research exceptions. Liability rules have been varyingly understood in doctrinal thought, for example as protection mechanisms stemming from the law of torts that involve a collective decision as to the value of the entitlement (Calabresi and Melamed 1972), or as purely bilateral bargaining based *ad hoc* rules that allow the concerned party to “take now and pay later” (Merges 1996). But liability regimes have also been viewed and studied as a mixture of these two approaches. Such mixtures can take the form of stand-alone entitlements that offer alternatives to purely exclusive property rights, through either unwound intellectual property entitlements or mandatory registration processes, which generally include automated licenses without the power to exclude and a specific modality of the “take now, pay later” rules (Reichman 2000). Building upon this latter work, we argue that this last type of liability regime can also be carved within codified yet unwound intellectual property entitlements, where different trigger points are established *ex ante* for compensation through prior user rights. That is why we consider that the defence mechanisms that have been built into IPR systems to protect follow-on users against infringement claims within a classical property-based entitlement regime also constitute liability rules that determine in effect allocations and split asset entitlements (Burk 1999). They are in this sense “prior user rights” or “codified liability rules” within a property regime, where either a compulsory license is set at zero royalty, or a trigger for compensation is defined *ex ante* by the legislator (Burk 2009). To show the contribution of this analysis, we use the lessons from universally acclaimed research on paradigm breakdowns in science (Kuhn 1962), to investigate whether, in certain areas of plant improvement technologies, a systematic series of breakdowns of the strong IP paradigm have appeared, and, if so, to examine a number of proposed institutional alternatives. To present the contribution of a systematic analysis of the relationship between institutional paradigms and their context of application to the construction of institutional and regulatory alternatives, the analysis in this paper will:

1. briefly review the shortcomings of the strong intellectual property rights paradigm in relation to certain technological evolutions, assessing the areas where this paradigm clearly contributes to agrobiodiversity-based innovation, and those areas where systematic or partial breakdowns are observed;
2. analyse and propose the adjustment of the intellectual property paradigm for the systematic and partial breakdowns witnessed in mass selection, controlled hybridisation and the development of molecular biology research tools.

## Shortcomings of the strong intellectual property paradigm as applied to plant improvement based on constant agrobiodiversity input

The design of alternative governance frameworks to promote the successful use of biodiversity in terms of efficiency, distribution and fairness, stems primarily from a single interrogation; that of the adequacy of the currently prevailing IPR paradigm, characterised by lenient patentability requirements and post 1991 enhanced plant variety rights, *vis-à-vis* all ranges of innovative and sustainable uses of agrobiodiversity. Responding chiefly to the needs of the increasingly intricate, knowledge-intensive, and incrementally cumulative context of agrobiodiversity research and development, dominated by molecular plant breeding and DNA recombination, the dominant strong IPR paradigm operates to the detriment of other existing innovation models, be it at community or farmer level, within both the public and private sectors. Our analysis is based on the premise that rules defining the contours of protection and use of plant-related innovations do not adequately correspond to the needs of the entire range of existing agrobiodiversity innovation systems, on account of the characteristics of seeds themselves, but also on account of the specific features defining plant innovation relying on the repeated use of agricultural biodiversity.

### Characteristics of seeds and plant improvement

Seeds embody an inherent duality, as they are not only commercial commodities in their own right, but they also constitute an instrument for technology transfer through their informational public good nature (Louwaars 2002), which farmers and breeders alike daily seek to improve. Seeds cannot in this regard be merely viewed as inputs for agricultural production, since the genetic resources they encompass also represent the key inputs of agricultural research and development activities, as potential sources of innovation. This feature has been enhanced through the infusion of science within traditional mass selection activities, which designate the long-established crop improvement techniques based on the observations, instincts and traditions of farmers (Gepts 2004). Even today, farmers from developing or developed countries alike develop new farmers varieties based on combinations of landraces, gene bank material and at times even improved varieties (Salazar *et al.*^2007^). Gradual scientific input into these mechanisms has led to the appearance of methodical and controlled hybridisation, based on empirical and systematic trial-and-error techniques, the detection of useful mutations and the 'fixation' of desired characteristics such as disease resistance or flavour enhancement through the deliberate crossings operated by plant breeders (Kingsbury 2009). The rediscovery of Mendelian genetics at the beginning of the 20th century, opening the prospect of unprecedented results in terms of yield, and introducing cultivators to ‘hybrid vigour’, has spread into the institutional structure of research and development activities themselves, gradually opening the doors to private investment in crop improvement (Jaffee and Srivastava 1994). The agricultural research paradigm then broke down once more, with the development of genomics science and our understanding of life at a deeper level, that of the gene (Buttel et al*.*^1985^). Even though most of the biotechnological advances witnessed in the early 1980’s have supplemented and reinforced the efficiency of conventional selection and hybridisation methods, they have moved the molecular base of new plant breeding forward in an astonishing way (Murphy 2007), while genetic modification and DNA recombination techniques have unveiled completely new horizons to the realisation of plant breeding’s promises (Kloppenburg 2004; Moose and Munn 2008). The two revolutions witnessed in relation to plant improvement, pertaining first to our understanding of genetics and rules of heredity, and second to the development of molecular biology and genomics science, have considerably altered the agricultural research paradigm and industry structure (Barton 2003). Scientific progress has indeed driven the rise of the private seed sector through the professionalisation of plant breeding, before igniting the reign of life-science giants through the infusion of molecular plant breeding capacity into the industry, alongside the vertical integration of technology-heavy start-ups.

As can be seen from this brief historical overview, agricultural biodiversity-related innovations first and foremost comprise a combination of knowledge disseminated through a wide array of informational tools and products produced by different actors. Crop genetic improvement is therefore essentially a process of derivation, whereby each incremental innovative contribution (realised either through the selection of best performing specimens on farms, through plant-breeding research relying on sexual or asexual crosses, or through various biotechnological instruments and techniques) holds the potential of becoming a commercial product. In this context, follow-on innovations or improvements directly derived from an underlying creation or invention raise, as in all "cumulative innovation chains", critical questions as to their degree of appropriability and control, and thus their optimal treatment (Merges and Nelson 1990; Lemley 1997). In the light of this peculiar innovation chain based upon incremental steps, rather than major leaps forward, steadily at the mercy of reverse-engineering, and the intrinsic public good nature of biodiversity’s informational component, regulatory intervention is inevitable for fostering investment in plant breeding, while ensuring the conservation of genetic variability. Such intervention needs nonetheless to be constructed around a particularly intricate balancing act, having due regard to past contributors to knowledge and biodiversity, as well as to future “borrowers” or developers of incremental knowledge and biodiversity, and thereby ensuring both prospects of appropriation and access to innovations. The present dominant intellectual property paradigm, characterised by extended patent protection and stringent plant-variety protection granted within a strictly regulated formal seed market, raises the question as to whether it has successfully achieved such a balancing act in the misunderstood world of agrobiodiversity innovation.

### Plant improvement and IP: Patents or sui generis Plant Variety Protection

The recognition of statutory property rights over the informational content of improved plant varieties was operated through the expansion of the scope of traditional IP mechanisms’ protection, as well as the enactment of need-specific protection regimes, so-called “legal hybrids” (Reichman 1994), derived from the same premises as the traditional protection regimes (Boyle 2003). The reality and reach of such proprietary protection climaxed through the adoption of international minimum protection standards in 1994 and Article 27§3b of the TRIPS Agreement, which quite uniquely starts with negative obligations, by stating that: *“Members may […] exclude from patentability: […] plants and animals other than micro-organisms, and essentially biological processes for the production of plants or animals other than non-biological and microbiological processes.”* Acknowledging the controversies surrounding the definition of patentable subject matter with regards to life sciences, the Agreement continues by asserting that: *“Members shall provide for the protection of plant varieties either by patents or by an effective* sui generis *system or by any combination thereof”,* thereby setting the foundations of the strong intellectual property paradigm in plant improvement.

#### Patent protection

By encouraging the inventor to disclose innovative knowledge, while simultaneously holding the rights to protect follow-on uses of inventions, patents avoid underinvestment in costly but socially beneficiary research and development activities, while also preventing the detrimental withholding of knowledge (Wendt and Izquierdo 2001). In so doing, they avoid some of the aggravated enclosure-related detrimental effects that might follow from recourse to trade secrecy (Eisenberg 1997). Within this context, patents, as titles conferring exclusive rights to use, sell and control the exploitation of a novel invention involving an inventive step and susceptible of industrial application, actually grant the right to “*prevent third parties not having the owner’s consent from making or using the product or process covered by the claim”* (Article 28§1 TRIPS), and are thus drafted as rights to exclude third parties. In accordance with Article 27§1 of the TRIPS Agreement, “*patents shall be available for any inventions, whether products or processes, in all fields of technology, provided that they are new, involve an inventive step and are capable of industrial application*”, subject to the exceptions to patentability set out by national legal orders. They can and have been granted intensively on biological material in accordance with the national patentability requirements where protection is sought, mostly in developed countries and in particular the USA. The extension of patent claims to the life sciences, mainly attributed to the infamous *Diamond vs. Chakrabarty* ruling of the US Supreme Court, had however been regular practice in a number of European countries from the 1930s onwards (Straus 2003; Van Overwalle 2006). The scope of patentability is today more intricately delineated, especially on the “old continent” (Europe), where plant varieties and essential biological processes are excluded from protection, in accordance with Article 53§b of the European Patent Convention as revised 29^th^ November 2000 (also present in the 1973 text). Nevertheless, even with the more recent recourse to subject-specific regulatory texts with relatively strong binding potency, such as the European Directive 98/44/EC on the legal protection of biotechnological inventions, defining the exact contours of patentability exclusions with regards to biological material has become an increasingly complex task. Indeed, the so-called “more than a single variety approach” upheld by the European Patent Office in 1999 (*Transgenic Plant/Novartis II* ) has been effectively extended by Article 4.2 of the aforementioned “Biotechnology Directive” 98/44/EC. While the EPO accepted that plant varieties could fall within the scope of a claim, if such claim did not cover a single plant variety, the Directive went on to assert that “plants, animals and their separate parts [were] eligible for patent protection” (Chiarolla 2011). Likewise, the difficulty to determine the impacts of enlarged patentability scopes on the access to essential research resources in various innovation chains is epitomised by the daunting proportions taken by recent attempts at patent landscaping (such as the CAMBIA rice genome landscape). These complexities, explaining the growing importance of legal counsel in order to navigate and enforce protection titles, stems mainly from the remarkable advent of biotechnology and the correlated expansion of innovative breeding processes or tools retaining a biological character, yet constituting important technological leaps forward, and therefore falling under patent protection, especially with regard to molecular selection efforts involving a number of highly technical and non-microbiological steps (Kock 2007).

#### Plant variety rights protection

A relatively lesser-known intellectual property-rights system (outside those having recourse to its instruments regularly), plant breeders’ rights (PBR) or plant variety protection (PVP), has been developed from 1961 onwards, with the enactment of the first Convention under the auspices of the “International Union for the Protection of New Varieties of Plants” (UPOV). The requirement set out in Article 27§3 of the TRIPS Agreement does not make direct reference to the UPOV system, even though today it seems to be considered as the main (if not the only) *sui generis* plant variety protection system that is duly implemented and complied with in an *effective* manner (Llewelyn 2004). PVP titles, as envisaged within the UPOV system, confer a bundle of rights to the developer of a novel *combination* of genes manifested as a distinct, uniform and stable variety, aiming therefore at the phenotype of the variety, rather than its genotype or its isolated biological components. These titles, offered under national or supranational legislation (as in the European Union through the Community Plant Variety Office), require neither proof of an inventive step nor a specific utility, as they are solely based on the evaluation of the variety’s value in terms of genetic quality, i.e. uniformity and stability, and on the basis of phenotypic differences *vis-à-vis* “known” varieties. The conditions for protection, formerly found in Article 6 of the 1961 and 1978 Acts with slightly different wordings, now state, in accordance with Article 5 of the 1991 Act, that “t*he breeder's right shall be granted where the variety is new, distinct, uniform and stable*”. According to Article 14 of the same instrument, the authorisation of the developer of the protected variety should be sought for “*the production, reproduction, conditioning for the purpose of propagation, offering for sale, selling or other marketing, exporting, importing and stocking*” of the propagated material. The exclusive rights granted by such PVP titles are however surrounded by two major counter-conditions, drafted in the shape of liability rules: the breeders' exemption and the farmers' exemption, evidencing the “tailored-for-purpose” nature of this protection mechanism.

The ***farmers' exemption***, allowing farmers to sow seeds for saving, using or exchanging, was in the past implied by the 1961 and 1978 Acts through the scope of protection granted to breeders (since the extent of exclusive rights did not reach acts perpetrated without any commercial purpose by third parties, including unmethodical selectors or farmers; Pires De Carvalho 2010). From such an exclusion from the scope of protection, the farmers’ exemption has evolved into a formal yet optional exception to the extent of the PVP title that may be granted at the national level for the use of seeds on the farmers’ own holdings and with possible equitable remuneration to the breeder, according to Article 15§2 the 1991 UPOV Convention. The ***breeders’ exemption***, granting plant developers the possibility to use protected varieties in their breeding programmes without prior consent from the title holder, had already been formulated in the 1961 text of UPOV. Article 5 stated that the prior authorisation requirement established “*for production, for purposes of commercial marketing, of the reproductive or vegetative propagating material, as such, of the new variety, […] shall not be required either for the utilisation of the new variety as an initial source of variation for the purpose of creating other new varieties or for the marketing of such varieties”*, except “*when the repeated use of the new variety is necessary for the commercial production of another variety*”; a wording that remained unchanged in the 1978 Convention. Article 15§1 of the 1991 text now provides for a “*compulsory exception”,* whereby the breeders’ right does not extend to *“acts done privately and for non-commercial purposes, for experimental purposes, and acts done for the purpose of breeding other varieties”.* The UPOV approach, as an instrument specific to the field of incremental plant innovation, thereby takes due account of the characteristics of seed development, and provides for a more adequate institutional fit to the specific features of conventional plant breeding. However, both exemptions have been significantly restricted in the 1991 text.

### Shortcomings of the property paradigm for addressing plant improvement

The inherently incremental nature of controlled plant improvement leads to the assertion that major leaps forward would be much rarer than in other more classical fields such as electrical engineering. Except for genetically engineered crops (transgenics), leaps achieved in agrobiodiversity innovation chains therefore need to be stretched to effectively qualify as "an inventive step" beyond the existing "prior art", and accordingly open the door for exclusive monopoly under the patent paradigm (Reichman 2003). The requirement of non-obviousness or of the existence of an inventive step found in all national patent systems thus theoretically prevents the patentability of plant varieties, unless the criteria for such patentability are revised or re-interpreted with lower standards (Barton and Berger 2001). As in synthetic biology, a sphere that draws inspiration from biotechnology, most of the innovations present in conventional or molecular plant-breeding innovation chains constitute in too many ways a novel recombination of already-existing components or varieties to be effectively protected under the historical patent paradigm, developed for the purposes of inanimate and chemical rather than self-replicating biological inventions (Rai and Boyle 2007). Nevertheless, even though the obvious character of most plant-related process innovations is frequently highlighted, the gene products of such methods may be considered as novel in the current patent paradigm characterised by lenient patentability requirements. This approach creates extensive objections before competent Courts and Patent offices on the damaging spectre of non-novel and broad biotechnology patents (Van Wijk 1995). Indeed, while the exclusive monopoly rights awarded to the initial developer by patent protection need to be “commensurate with the contributions to the state of the art” (Straus 2003), such contributions may not be easily determined in those plant improvement models that rely on biological processes. Drawing on the incremental nature of plant innovation, the pivotal notions of prior art and novelty or non-obviousness carry further cravings for vigilant consideration, as the currently dominant intellectual property paradigm is considered to have enclosed what is by definition not enclosable in cumulative innovation processes, while failing to duly recognise past contributions, small or big, of previous germplasm users and conservers, urging calls for caution as to the possible "recycling of public knowledge for private reward" (Drahos and Braithwaite 2003).

When analysing the magnitude of the rewards that can be attained through innovation, especially within a cumulative cycle with few ground-breaking discoveries, it should also be remembered that actors possessing restrictive monopoly rights have the ability to "choose the optimal level of output for the intermediate good embodying the patented technology" (Goeschl and Swanson 1999). The proliferation of strong and broad foundational patents, designating not only one technological application but encompassing a range of claims, is therefore thought to impede the entire research community's range of action (Salazar *et al.*^2000^). This could in turn potentially threaten an innovators' inherent right to build upon another innovators' creation, and tamper with the intricate balance between the appropriation and diffusion of innovations. As a consequence, fluctuating and fragmented patent landscapes significantly increase the known and unknown costs of research and development, all the while creating “a great deal of uncertainty in making product development and investment decisions, which rely on a realistic ‘freedom to operate’ assessment", both within the private and the public sectors (Henkel and Maurer 2009; Chi-Ham et al. 2010). Furthermore, when competing firms hold patents on different components of a complex technology (thereby creating a phenomenon that has been denoted "a patent thicket"), and decide not to cross-license them, research and development activities can be slowed down or even rendered impossible in an entire industry (Shapiro 2001). Examples regarding delays in attaining research results, or simply conveying the difficulties in gaining access to technologies or a small part of a complex technology are regrettably numerous (Kingston 2001). Within the context of transgenic research, we may cite the setbacks experienced by the relatively large American Cyanamid (since then acquired by BASF) in product development due to the exclusive licensing agreement signed by the "Biolistic Particle Delivery System" gene gun technology developer, Cornell University, towards the university researchers' own start-up company, Biolistics, which was later bought by DuPont. Negotiations between the two companies failed, partially due to their competitor status in a different product market, causing considerable delays in Cyanamid's alternate product development cycle (Pray and Naseem 2005). Patents in life sciences have indeed sometimes been used as trading currencies or bargaining chips, as defensive means to prevent lock-outs caused by a competitors’ denial of access to its invention, in contrast to simple technologies such as chemicals (Merges and Nelson 1990; Kingston 2001). Another infamous example of so-called “blocking patents” on complementary technologies, covering broad market segments and heavily affecting new entrants (Merges 1994), relates to the development of pro-vitamin A-enriched ‘Golden Rice’. This variety, developed upon a public-domain premise through the initiative of the Rockefeller Foundation, required permission with regard to about 70 patents in the United States, widening concerns *vis-à-vis* the sacrosanct "freedom to operate" in biotechnology-backed plant-breeding activities, even though the patents were seemingly relinquished in favour of the poor in this particular case (Kryder *et al.*^2000^; Hope 2008).

In response to the potent need for effective protection of innovative products, faced with the daunting prospect of rapid reverse-engineering, especially in the case of the transgenics innovation system, the proprietary paradigm has thus been established and gradually extended by public authorities to address forms of innovation that were not traditionally protected under patent-like regimes, such as those stemming from conventional or molecular plant breeding. However, these extensions reflect a generalised and pervasive contraction of artificial lead times in cumulative incremental innovation processes, thereby straining the regulatory system to its breaking point, and weakening the competitive ethos upon which intellectual property rights continue to be based (Reichman 1994).

## Institutional alternate tools: paradigmatic fit and responses to breakdowns

Acknowledging the shortcomings that emerge from the excessively restrictive and exclusive rights embodied in the strong intellectual property paradigm, the limits around biodiversity use, appropriation, production and re-use need to be clearly re-defined, taking due account of the incorporeal nature of relevant innovations, the ease of reverse engineering, and the colossal investments required for new developments. Systems drawing from completely open-source models or from such understanding would in this regard probably fail to deliver significant portions of socially, agronomically or environmentally meaningful innovations. Far from the pleas that have been associated with equity or piracy discourses, alternatives should be developed, recognising the importance of the rules of access to innovation which characterise intellectual property regimes, but also acknowledging the need to reward the contributions of both previous and future stewards and users of agricultural biodiversity. Conceding that no perfect trade-off between protection, access and diffusion can exist, the rights, privileges and use conditions inherent in the current patent and PVP approaches may still be adequately distributed in a more coherent regulatory framework which would attempt to address the needs of all sub-systems of agrobiodiversity-reliant innovation.

Considering the flexibilities in the TRIPS Agreement, i.e. the principle of an “*effective sui generis plant variety protection system*” mentioned in Article 27§3, the recognition of exceptions from patentability and protection scope, *inter alia* in Articles 27, 30 and 31, and the room provided for the regulation of licensing protocols (WIPO, World Intellectual Property Organization 2011), we believe the task at hand hinges on experimenting in a more consistent manner with various models that build partially-open innovation systems based on such flexibilities and on second tier liability rules. In this article, we aim to show the contribution of such a complementary framework for agrobiodiversity-based innovation, by looking in a more consistent way at a set of tools that may address the cases of systematic breakdown (witnessed in Mass selection) and partial breakdowns (where both proprietary and partial openness driven institutional approaches may prove valuable, more specifically in Controlled hybridisation*,* and Molecular biology upstream research tools).

### Mass selection

#### Cultivar selection and exchange on the margin of the strong IP paradigm

Farmers have been compelled to radically rethink their traditional production chain in the light of the drastic changes occurring in agricultural production, first through the technological revolutions that have instilled new high-performance inputs on farm, and then through novel regulatory frameworks that have constructed ‘artificial’ boundaries on the use of such inputs. Farmers had previously reproduced their seeds time and time again, and exchanged those warily selected best-performing or best-fitting varieties with other farmers. Even today, a large proportion of the seed planted worldwide is either saved by farmers or exchanged on a farmer-to-farmer basis. In the mid-1980s farmer-saved seed accounted for an estimated 35 per cent (or $18 billion) of the total estimated value of $50 billion of all agricultural seed used worldwide, proprietary or not (Groosman et al. 1988). In developing countries, the importance of seed-exchange networks and re-use is seemingly even greater, as an estimated 80 per cent of the seed used in the early 1980s was farmer-saved (Pray and Ramaswami 1991). In addition to the main vertically integrated innovation chain producing improved varieties, mass selection operated on farms by farmers cannot thus be overlooked as an innovation system contributing to the conservation and sustainable use of agrobiodiversity, whilst also truly ensuring the subsistence of millions of farmers based on principles of open access and informal exchanges. Mass selection operates on a daily basis even in developed nations, where a number of noteworthy initiatives have emerged. The French network "*AgroBio Périgord, Maison de la Semence*", for instance, disseminates a technical book on the multiplication and selection of maize and sunflower on farms to the 250 growers that take part in the Western France network. In order to conserve non-proprietary agricultural biodiversity, they experiment on local populations or 'landraces', selecting those individuals presenting similar characteristics after two or three years of natural local adaptation, without ever falling under a stock of 600 individuals (in order to avoid degeneration and maintain so-called "security stocks").

However, such selection and exchange networks need to re-situate themselves within the technological and regulatory environments that currently surround their activities, if they wish to sustain their endeavours without infringing other actors' intellectual property rights. They are also faced with an additional complexity created by the legislation surrounding the rights to distribute seeds in general, whether these are protected through IPR or not. This age-old innovation model is indeed not only set aside by the dominant appropriation and incentive mechanisms set out by IPR tools, but it is also pushed into illegality by those rules solely pertaining to the regulation of the “formal” seed market. Indeed, in most developed countries, and increasingly in developing nations, seed distribution is only allowed after certification procedures based on the distinctness, uniformity and stability of plant varieties (so-called DUS testing), and the inclusion of either actors and/or varieties into official catalogues (Tripp and Louwaars 1997). While formal seed markets function on the basis of regulation pertaining to approval and promotion, with quality insurance and guarantee as to the identity, purity and performance of purchased seeds, informal exchanges are governed by cultural norms and *ad hoc* rules determined solely by the participants in the exchange, without regulatory intervention (Lipper et al. 2009). Yet mass selectors are forced into illegality by both seed marketing requirements and IP rights, while not being compensated for their efforts to cultivate and upgrade biodiversity on farm. The possibility of farmers/selectors infringing upon existing patent or plant variety rights remains extremely high, depending on a wide range of circumstances relating to the national legislation in question, as well as to the specific crop or the size of the farming enterprise. These factors influence the recourse and range of the farmers’ privilege recognised under PVP laws. In addition, the recourse to a wide array of enclosing instruments by innovators for a single specific product (such as Round Up Ready canola), simultaneously protected through process and gene patents, plant variety rights, trademarks and the private contract that is the “Technology Use Agreement”, has further complicated the delineation of the farmers’ privilege. The shrinking space for manoeuvre left to farmers for seed saving, using and exchanging within the strong IP paradigm, and a seemingly generalised lack of awareness or training in legal issues on the cultivators’ side, have led to mounting disagreements between variety developers and sowers, leading to numerous court cases. Litigation has flourished over a range of IP infringements, from the possession of protected seed in itself, to its re-use outside the scope of the legislation, without royalty collection or in larger farms than those targeted by applicable legislation. Furthermore, mass selection has been increasingly tainted by illegitimacy indictments through stringent formal seed market regulations, aimed at ensuring seed quality, consumer protection and high productivity rates. While farmers are protected against crooked seed distributors and impure seed lots by the establishment of official catalogues and clear insurance or liability regimes, the varieties they would perhaps want to cultivate for agronomical, environmental or cultural reasons fail to meet the requirements of the stringent marketing rules of distinctness, uniformity and stability. Litigation has in this regard prospered, especially over the lack of equivalent certification requirements for the commercialisation of conservation varieties, where the lack of registration of farmers’ varieties in national catalogues has generally been ruled to be in violation of the formal seed market rules (*GNIS and FNPSPF vs. Kokopelli*), and has recently been seen as unfair competition (Tribunal of Grand Instance of Nancy, *Graines Baumaux vs. Kokopelli*; cf. detailed discussion below).

Not only does the dominant paradigm push mass selection activities into the realms of compulsory illegality, it also systematically fails to equitably acknowledge mass selection networks that are not given efficient tools to protect the products of their activities and foster their own innovation model based on open access. Indeed, artificial lead time is not provided to this model, thereby failing to address the market failures with regards to this specific innovative process (Reichman 1994). Even though traditional exclusion rights embedded in IP tools do not adequately fit the collective nature of innovation based on mass selection, the diverse and unstable, yet locally adapted varieties ought to be protected against subsequent re-appropriation by either other farmers or the industry, and compensation should be triggered, in case of re-appropriation for instance, at the commercialisation stage of new varieties incrementally developed by third parties. The ability of farmers to develop new varieties based on mass selection is regrettably still largely ignored by policy-makers, confronted with a regulatory conundrum where protection should not only be granted to the conserved germplasm or created material, but also appreciate the farmers’ “dynamic and collective system of technology development and diffusion through every season”, based on skill sharing and seed exchange (Pelegrina and Salazar 2011). The institutional characteristics of mass-selection efforts therefore point towards a systematic breakdown of the strong exclusive-appropriation-oriented paradigm with regard to this less technology-driven and more community-oriented innovation system.

#### Models responding to the paradigm breakdown for mass selection

Mass selectors are faced with the double challenge of shrinking possibilities to re-use protected improved varieties under more stringent PVP and patent legislation on the one hand, and of shrinking possibilities to exploit their own uncertified farmers’ varieties in the formal seed market on the other. As argued in this section, they may find solutions in either farm saved seed regulation or in derogatory certification schemes, which may include a second tier liability rules regime.

##### A. Farmers’ exception and farm-saved seed regulation

Until now, the main approach to allowing mass selection-based innovation to contribute to the sustainable use of biodiversity on farms has been to situate the “traditional practices of farmers as exceptions to the exclusive rights of plant breeders under existing IPR tools”. This precludes breeders from demanding payment from farmers who save and plant seeds saved from prior purchases, or informally exchange purchased seeds (Helfer 2002). The farmers' exception or privilege, allowing farmers to sow seeds for saving, using or exchanging without the authorisation of the variety developer, as it was defined in the first UPOV Conventions, allowed for farmers/selectors to use the diversity created by breeders in their own selection routine. Although the 1978 Convention, being a minimum standards agreement, granted opportunities for the more precise design of the implicit rights’ contours at the national level and thereby more greatly limited non-commercial uses, it should still be noted that under the practice of so-called “brown-bagging” in accordance with this Act, farmers were even allowed to sell limited quantities of protected seeds for reproductive purposes (Ghijsen 1998). Today, this privilege, which could either be viewed as an exemption from infringement or as an exception to the variety developer’s rights, has become formally conditional to elements related to national circumstances, farm size and the necessity to use the seed on the same farm, and has also been surrounded by licensing obligations (Dutfield 2008). Indeed, the formerly implicit exemption is now enshrined in Article 15§2 of the 1991 UPOV Convention, which states that “*each Contracting Party may, within reasonable limits and subject to the safeguarding of the legitimate interests of the breeder, restrict the breeder's right in relation to any variety in order to permit farmers to use for propagating purposes, on their own holdings, the product of the harvest which they have obtained by planting, on their own holdings, the protected variety*”.

The wording clearly shows the shift in approach to the farmers’ exception, which evolved into an *optional exception* to the exclusive rights of breeders, rather than an array of actions considered outside the scope of the IP title in itself. Such restrictive evolution has been indicated to be the source of the recognition of farmers’ rights within the FAO system as a bundle of socio-economic rights including those related to seed as such (as asserted by Article 9 of the International Treaty for Plant Genetic Resources for Food and Agriculture, entered into force in 2004; Pelegrina and Salazar 2011). Landmark cases in the USA and Canada have reiterated that the farmers’ exception should be interpreted in a narrow fashion *vis-à-vis* the sale of the protected-varieties’ progeny (such as the ruling in *Asgrow vs. Winterboer* that identified “brown-bagging” as a marketing practice violating modern United States’ legislation)*.* The European legislation has, in a parallel fashion, dressed the contours of farm-saved seed quite restrictively, and especially conditioned the farmers’ privilege to the payment of “an equitable remuneration […] sensibly lower than the amount charged for the licensed production of propagating material” in Article 14 of EC Regulation 2100/94 on Community Plant Variety Rights. This Article further imposes an information obligation on the farmers and suppliers of processing services *vis-à-vis* farm-saved seed quantities. Both national and European case-law has been built around the interpretation of such terms, balancing the interests of farmers and those of breeders with those human rights to privacy and avoiding abuse of rights on all accounts. National rulings have for instance protected farmers’ interests against systematic invoicing for seeds that are saved but not used for sowing or multiplication purposes, and against the gathering of farm-saved-seed information without the consent of farmers. They also pointed against potential abuse of rights through the lack of recourse to other available remedies for information collection, and the invalidity of claims issued without any indication of possible infringements, especially the intent to sow the product of the harvest for propagating purposes, as asserted by the European Court of Justice in *Schulin vs. Saatgut* (Case C-305/00, 10 April 2003). The French national order has in this regard shown the strictest reaction to the privilege, establishing it solely with regards to wheat through a voluntary compulsory contribution system and considering all other farm-saved seed to be counterfeit, falling within the realm of law 2007–1544, dated 30^th^ October 2007. These various legislative developments push oneself to question whether and to what extent the farmers’ privilege should be recalibrated in order to maintain its initial rationale and ensure the survival of mass selection endeavours.

The inherent concern of the PVP system *vis-à-vis* farmers is not omnipresent within patent laws as such, as these statutes tend to remain abstract in their nature and are therefore not designed to solely apply to agrobiodiversity-reliant innovations as PVP legislation is, with its inevitable links to the loftier issues of food security or environmental protection. Certain countries have nonetheless equipped themselves with a number of legislative tools in this regard, such as the case of European legal order and its Directive 98/44/EC, making room for a farmers’ privilege in domestic patent systems in its rather unusual Article 11§1, allowing farmers to retain material grown on their own farms for subsequent years (Nenow 2001). While this instrument has no direct effect in Member States’ national legal orders and allows for restrictions of these rights awarded to farmers, it still acknowledges the specificity of the socio-technological innovation system that is mass selection. Even though the so-called “Doha round” and its Ministerial Conventions have seemingly failed to fashion a viable consensus on the terms of a new World Trade order, they have strengthened regulatory determination to include such privilege within domestic patent regulation, leading for instance to the 2007 amendment of the Swiss Federal Patents Act so as to include a farmers’ privilege, limited to uses of the patented material within the same farm (Pires De Carvalho 2010). The relatively rare recourse to the exception within patent legislation could be entrenched in a textual TRIPS interpretation, in accord with which the recognition of such a privilege to farmers might prejudice the legitimate interests of the monopoly holders under Article 27 (Watal 2000). The feasibility and conformity of such exceptions in patent legislation has yet to be tested before the judiciary or the WTO dispute-settlement mechanisms, but we believe that the flexibilities inherent in the Agreement and its rationale allow for the recognition of the farmers’ exception. Other commentators have in this regard highlighted the possibility that the existence of compensation in return for the right to use, save and exchange the protected material might actually encourage the doctrine of compulsory licensing, viewed as a “statutory license”, rather than as a classical exception to IPR protection as grounded in Article 30 of TRIPS (Garrison 2006). Furthermore, the reluctance to adopt formal farmers’ privileges in patent laws themselves can be overturned through jurisprudential liability thresholds, especially if plant-breeders’ rights recognise growers’ right to save and exchange seeds, as established before Canadian courts. Even though the patent-infringing canola farmer could not benefit from the privilege enshrined in PVP legislation to save the seed, monetary compensation deriving from the infringement was overturned on the grounds that no financial or other benefit was generated by the technology (Phillips 2007, analysing *Monsanto vs. Schmeiser*). This argument could fuel the debate on the liability thresholds that might be introduced for re-use conditions.

##### B. Reward regime for uncertified seeds and the legal status of exchange platforms

The outlines of protection regimes for mass selection and landrace revival networks should be assessed in general terms but also with due regard to national specificities, in order to safeguard centuries-long seed-saving and exchange practices, distancing oneself from the reductionist perception that farmers merely cultivate biodiversity developed off-farm by breeders. National regulatory frameworks should therefore consider innovation stemming from mass selection as a parallel yet different (and not necessarily derogatory), seed-production scheme, raising different predicaments than dominant vertically integrated molecular plant breeding, and requiring incentives within a dual *sui generis* system for both modern and farmers’ varieties. Such system, taking due account of national or regional stages of rural and economic development, may counter the trend to limit mass selectors’ activities to the realm of exceptions, reducing farmer’s privileges to a “basic trickle of rights” (Cullet 1999). At this stage, the different options lie within seed marketing legislation on the one hand, where DUS requirements in certified seeds could either be relaxed, or derogatory *ad hoc* regimes for local varieties may be established; and within IP legislation on the other, where the UPOV system may be redesigned to allow for landrace protection and use, or where a set of minimalistic double tier liability rules may be further put to use in order to compensate farmers for the use of their varieties in commercial breeding programmes, relying perhaps on a “light registry” to track varieties down, as developed below.

While intended to standardise crop names, protect consumers and foster investment in breeding, existing mainstream certification legislation and market regulation have had “the unintended consequence of drastically reducing the numbers of cultivars grown and impinging on the ability of farmers to grow older varieties or landraces” that do not fit within the formal seed market (Vetelainen *et al.*^2009^). Alongside the rather weighty choice of relaxing the formal system so as to include a wider array of plant varieties and actors, the establishment of derogatory *ad hoc* regimes in the form of book logs or flexible national (or regional) registers of uncertified seed could be options worth considering. In Brazil, the recognition of mass selection operated for instance solely through amendments of the seed marketing legislation in 2003, where landraces or traditional varieties have found new legroom, notably through the possibility that “family farmers” have been granted to register landraces in the National System of Plants and Seeds. This registration includes specific criteria taking the cultural and traditional aspects of the varieties into account, without prejudice to the exchange possibilities in the absence of such registration, since an official double exemption from registration has also been foreseen (Santilli 2012). On the other hand, the establishment of a derogatory “light catalogue” has been the way forward in the European legal order, through Directives 2008/62 and 2009/145 on conservation varieties, to the dismay of both commentators and politically active farmers’ associations (Anvar 2008). Several research projects were funded through the FP6 European Research Framework, known as Farm Seed Opportunities, in order to overcome the inherent difficulty of uniformly regulating quite diverse farm-innovation systems. Targeted to support the implementation of seed regulations on conservation varieties, these projects also proposed complementary seed-regulation scenarios, the utility and effect of which may need further consideration. To all intents and purposes, opening the Catalogue to conservation varieties remains a means of reducing genetic erosion and preserving varietal heritage, even though critics have argued that such move entails the risk of undermining the main commercial system, and may potentially block completely open marketing possibilities for non-ind ustrial models of agriculture such as organic farming or bio-dynamics in light of additional administrative obligations (Bocci 2009).

In the absence of such “light catalogue” or a general exemption from certification, and thus in absence of a legal recognition of seed exchange platforms, litigation between formal and informal seed market actors will prosper, as shown by the aforementioned French case opposing *Kokopelli* to *Graines Baumaux*. Referenced by the Court of Nancy in February 2011 (Case C-59/11), the European Court of Justice needed to assess whether seed catalogues violated principles of the *acquis communautaire* related to the liberty of trade, free movement of goods, proportionality, equality and non-discrimination, as well as the Union’s obligations under international law, especially with regards to the Convention on Biological Diversity and the FAO International Treaty. The opinion of Attorney General Kokott, issued on 19^th^ January 2012, seemed to indicate that the International Treaty did *“not include any provisions which are unconditional and sufficiently precise as to challenge the validity of EU legislation on the marketing of seeds*”. However, in the light of the proportionality principle*, “the disadvantages of the marketing prohibition, [which include a negative impact on the freedom to conduct a business and agricultural biodiversity] manifestly outweigh its advantages”*, a disadvantage that is not sufficiently attenuated by the derogations carved out by Directive 2009/145. Indeed, the advocate general argues that the conservation varieties Directive, by not giving “*sufficient consideration to the interests of economic operators and consumers”*, does not allow for sufficient scope *vis-à-vis* the use of old varieties and those products of mass selection, thereby concluding that “*the prohibition on the sale of seed of varieties that are not demonstrably distinct, stable and sufficiently uniform […] invalid as it infringes the principle of proportionality, the freedom to conduct a business […], the free movement of goods […] and the principle of equal treatment*”. The judgment of the Court, issued on 12^th^ July 2012, ran counter to the initial conclusions set out by Attorney Kokott, by taking a rather positivist approach to the principle of proportionality within the European *acquis communautaire*. In this regard, the Court assessed whether the exclusion of non-distinct, stable and uniform varieties from the formal seed market was appropriate for attaining the legitimate objectives pursued by official catalogue legislation. These objectives were identified as the increase of agricultural productivity and the reliability of the characteristics of the seed, which were adequately pursued by the litigious measures, setting the grounds of an efficient market without completely ruling out the marketing of old varieties. The decision should be analysed in light of the fact that the conservation varieties Directive was not in force at the beginning of the proceedings, although the national Court has been invited to take account of such legislative development. Indeed, the Luxembourg based Court seems to hint that the forced move of *Kokopelli* into illegality and unfair competition has been remedied by the enactment of this *ad hoc* regime. Were it to be the case, the imposition of geographical, quantitative and packaging restrictions by the derogatory rules of the “conservation varieties” catalogue would seemingly not undermine the recognition of mass selection efforts, according to the ECJ’s approach to the issue, although it might actually minimise their impact in practice.

Even though the illicit or uncertain legal status of exchange platforms ought to be remedied, a wider regulatory debate is also needed on the form of a reward regime for the development and maintenance of farmers’ varieties, which would satisfy both mass selectors and methodical breeders. The International Law Association Committee on the International Law on Biotechnology suggests "examining whether the UPOV system should be partly adapted and relaxed to allow protection of improved farmers' varieties that result from controlled on-farm breeding processes" (ILA, International Law Association 2010). However, amending principles related to protectable subject matter in the current property paradigm would imply radical changes in rationale and attitude, notably because of the inherently variable, non-uniform and collective nature of farmers’ varieties (Correa 2000). Indeed, the subject matter requirements of the existing strong-IPR approach relate to new and clearly distinguishable plant varieties, and thus “often cannot accommodate the contributions of individual farmers using more informal methods to select for better crops or sought-after plant characteristics” (Helfer 2002). The recognition of biodiversity-related collective intellectual property rights in the hands of local communities has, for instance, been pushed forward within the Indian legal order, within a system where property rights are shared with governmental authorities in an attempt to fill the gap in perception and ensure compliance (Cullet 1999). If a parallel protection regime for uncertified farmer seeds is privileged, special attention should thus be given to its contours, especially with regard to equity concerns. Indeed, protection should only concern varieties in themselves, and not extend to their genotype, as it should allow for the acknowledgement of the efforts lying behind mass selection, which are often collective, while also reflecting on the adequacy of the “exclusivity” approach within such communities, where open licensing and remuneration systems might prove better-fitted (Correa 2000). Indeed, regulators should take notice that the allocation of exclusion rights to mass selectors refutes the rationale upon which this rather unique partially open innovation system is built. Conversely, the design of a reciprocal liability rules mechanism might prove effective in providing compensation to selectors in case their varieties are used in commercial breeding programs and subsequently marketed. The aforementioned solution in seed marketing legislation, i.e. the constitution of a “light registry for farmers’ varieties” could for instance be the starting point of such a minimalist liability rules regime, providing not only some artificial lead time but most importantly a modest royalty rate to be forfeited by variety borrowers (Reichman 1994, 2000), similar to the “equitable remuneration” perceived by patent or PVP right holders on farm-saved-seed. Such option would however need to contemplate the tricky issues of designating the rightful interlocutor, addressing whether similarity or “substantial difference” thresholds should be established in the assessment of follow-on innovation, and whether litigation should be avoided through mediation mechanisms, or whether compensation should be integrated into institutional frameworks such as participatory plant breeding schemes where contractual reward for subsequent commercial use could be envisaged at the start of the project.

Ensuring the production of public goods that are the result of mass selection thus entails a comprehensive fine-tuning of dominant intellectual property regulatory tools, reconsidering the existing flexibilities aimed at *in situ* biodiversity conservation within the strong paradigm. Mass selectors are not only pushed into illegality through the shrinking practices of farm seed-saving, but also through the uniform regulation of the seed market and all correlated legislation establishing official catalogues ensuring the quality of seed circulating in markets and the seriousness of actors involved. The farmers’ exception and those regulatory takes on farm-saved seed should be recalibrated in PVP legislation and provided for in patent laws, while, most importantly, a well-suited reward regime should be constructed to free mass selection and exchange practices from the present illegality, thereby ensuring the maintenance and continuous production of biological diversity upon which breeding programmes continue to rely.

### Controlled hybridisation

#### Incrementally sequential innovation on the margin of the strong IP paradigm

The development of science-based plant breeding is marked by the shift from unconscious mass selection towards conscious rational attempts at adaptation, characterised by the use of the Mendelian principles of heredity and segregation, which led to the development of controlled hybridisation, relying upon inheritance-focused methodical selection anchored to the phenotypic observation of plant varieties (Bowler 1989). Through the lengthy empirical study of mutations obtained through deliberate crosses of plant varieties, breeders can predict the advent of specific characteristics such as drought resistance or fungal tolerance. A number of varieties may also notably outperform the parental lines used in their development, through the less-well-understood principle of heterosis or hybrid vigour. It is mainly this phenomenon that has truly transformed breeding into the “lucrative science” we observe today (Fowler 1994), as the advantages conferred on new varieties by hybridisation methods cannot be replicated in farmer-saved seed (Evenson 2005). Controlled hybridisation is characterised by long cycles of research activity before the commercialisation of the end product (i.e. the improved variety), with the initial two years of breeding programmes focusing on the deliberate production of mutations and variety crosses. This is followed by the “lengthy and tedious” selection stage, with six to eight years devoted to the examination of the best recombination and stabilisation designs for the new variation (Van Den Hurk 2009). Acknowledging that "exotic germplasm" may at times make all the difference between competitors' similar products, conventional plant-breeding activities still rely heavily on the largest possible gene pool of improved varieties, while successfully marketed varieties constitute the backbone of conventional breeding programmes. Breeding programmes are therefore heavily affected by the strengthening of intellectual property rights through extensions of the scope of protection regimes and ever-growing limitations surrounding the possibility of re-using the protected product, process or variety.

In order to balance and better enforce the PVP system and provide more exclusivity through a tougher stance on “plagiaristic breeding”, a novel concept, that of essential derivation was developed in 1991 to extend the range of acts that would require a breeder’s authorisation. The 1961 and 1978 acts were deemed insufficient for this purpose in the light of the globalised industry’s needs (Wendt and Izquierdo 2001), in order "to prevent converted lines from infringing and pirating breeder's genetic material" (ISF, International Seed Federation 2005). Under UPOV 1991, the necessity to negotiate a licensing agreement not only emerged in cases where the protected variety's use in a breeding programme led to the commercialisation of a new variety that is not clearly distinguishable, but also in cases of an "essentially derived variety" (EDV), defined as one "*predominantly derived from the initial [improved] variety, retaining the expression of the essential characteristics that result from the genotype or the combination of genotypes of the initial variety*" (Article 14 paragraph 5 of the 1991 UPOV Act). The trigger point for authorisation thereby shifted from distinctiveness to the determination of essential characteristics. The main challenge faced by conventional plant breeders in this regard relates to the shrinking room for manoeuvre left for the use of protected material in breeding programmes. Even though the “absolute permission rules” still delineate informational property titles (in accordance with which the permission of the monopoly-owner ought to be sought for using the protected information), liability rules (whereby the entitlement can be used without permission so long as adequate compensation is granted later), embody the specificity of cumulative plant-breeding innovation through the unequivocal breeders’ exemption of PVP legislation (Merges 2001). The concept of essential derivation was not designed to weaken such exemption, but rather to fight plagiarism. However, the wording's vagueness, as well as the interpretation it received in practice as a tool to be used against all varieties tenuously resembling and thus directly competing with the "initial variety", meant that it bore the perilous risk of reducing the nature of statutory undeniable-use exemptions. Such an extension retains a disquieting potential as a weapon to shut down or delay competitors possessing potentially better-performing yet dangerously similar products (such as the relatively constrained Mediterranean market for brown tomatoes for instance), rather than trying to identify infringers, thereby creating unnecessary and unproductive hostility within an innovation chain that is bound to create similarly derived products. Therefore, with little consensus over the genetic conformity threshold required to activate this extensive protection granted to cosmetic modifications, the EDV addition could be detrimental to small-scale breeders (Narasimhan and Robinson 2008). Rather than granting breeders more protection, the new balance found in the international breeders' rights legislation might thus overlook the role of liability rules embedded within the breeders’ exception in the successful use of agrobiodiversity on farm and other small breeding programmes.

In parallel to such newfound reach in PVP legislation, conventional breeders increasingly began to be confronted with an unfamiliar and strong legal entitlement, i.e. patents, where relatively restricted room has traditionally been awarded to follow-on use possibilities *vis-à-vis* protected innovations, especially in active breeding programmes. Indeed, patent legislation worldwide extremely rarely provides for exceptions to exclude third parties with specific respect to research conducted within the protection innovation, or to breeding. However, companies that still generate their income from plant-variety licensing, sale or distribution, rather than patented biotechnology research tools or process licenses, continue to unreservedly rely on the accessible nature of both improved and exotic agricultural biodiversity (Louwaars et al. 2009). The restrictions stemming from strong rules of appropriation with regard to the accessibility of improved genetic material, while preserving the positive prospect of royalty income, may also hamper the sacrosanct “freedom to operate” that breeders long for. Increased opportunities for exclusion, granted at the phenotypic level through plant-variety rights and at the genotypic level through patents, cannot thus wholeheartedly be considered as vehicles for fostering innovation with respect to plant improvement. This leads to a partial paradigm breakdown with regard to conventional plant-breeding activities in terms of follow-on possibilities. The recent turmoil created by the support given, especially in the European continent, to the recognition of a breeders' exemption within patent law, and for an extension of the existing exemption under UPOV-like plant-variety protection to the commercialisation stage, coupled with the calls for better defined and balanced public/private research partnerships, show the existing disquiet about the future of all agricultural research and development.

#### Models responding to the paradigm breakdown for controlled hybridisation

The tensions that have arisen “between first generation breeders who have secured legal protection for new varieties and second generation breeders who seek to utilise those new varieties to develop more varieties” need to be duly addressed so as to continue to permit second generation innovators to engage in the production of public goods (Helfer 2002). The breeders’ exemption, which stands out as an efficient prior user right that could be associated to an *ex ante* liability rule operating under a “take now, pay later” understanding codified so as to allow both the use of protected material and the compensation of the initial plant breeder (Merges 2001; Burk 2009) should be advocated further, as a response to the shortcomings of the strong IPR approach. The threat to such an exemption in PVP systems should be carefully addressed, notably in the light of the EDV concept, and also of the inevitable co-existence of PVP with patents on the same material or within the product development chain as a whole.

The contribution and opportunity of a breeders’ exemption in patent regulation should be further assessed in the light of the characteristics of the technology development mechanism. While those breeders' exemptions recognising immediate rights over protected material for further use in breeding programmes remain the absolute foundation of plant-variety-rights protection worldwide, these remain scarce in patent legislation. Under the tight-lipped TRIPS framework and the worryingly mute European Directive, merely a handful of national legal orders allow for breeding-specific research possibilities outside negotiated licenses. In Germany, France and Switzerland (and probably soon in the Netherlands), the infamous PVP breeder’s exemption has found its echo in patent legislation, where breeding programmes could be initialised, even when the material contained patented traits, the consent of the patent holder needing to be sought at the commercialisation stage. As a result of this exception, breeders may indeed use the protected variety in their crop genetic improvement activities (in accordance with the characteristics of science-based plant breeding discussed above), relying on constant and unconditional access to improved germplasm found in the market to generate socially, economically or environmentally interesting new agrobiodiversity on the basis of existing genetic variability. The lack of consent from the rights holder for carrying out active commercial research using the innovation is at first sight quite a positive departure from traditional patent protection, but its efficiency still needs to be tested (Blakeney 2011). Early indicators show that such flexibility has, in practice, resulted in hostile reactions from competitors wishing to shut down ongoing research activities (PLANTUM Dutch Breeders' Association 2009). There are also extremely heated discussions on the extent that the breeders’ exception should have in practice, and the trigger point where licensing negotiations would need to be undertaken, if the patented element should still be present at the stage of commercialisation or not. Furthermore, due attention should also be given to these negotiated licensing terms in order to ensure that socially beneficial innovation, in the form of better performing or adapted varieties, is distributed and not locked out by unacceptable conditions set out in a highly competitive marketplace.

### Molecular biology upstream research tools

#### Upstream research tools for plant breeding on the margin of the strong IP paradigm

Scientific breakthroughs achieved up to the middle of the 20^th^ century in genomics science by brilliant minds such as Alfred Hershey, Martha Chase, James Watson and Francis Crick with regard to the role and structure of deoxyribonucleic acid (more commonly known as DNA, a molecule discovered as early as 1869) have revolutionised the agricultural research and development cycle through the development of biotechnology and molecular biology tools. A far-reaching term, "biotechnology" covers an extremely wide range of innovations, including industrial fermentation and modern, post-DNA-discovery genetic engineering, and has expanded the science of plant breeding through very efficient novel screening tools and variety development techniques. In doing so it has altered once again the management of agrobiodiversity in terms of conservation and use possibilities, as well as the landscape of actors involved. Research tools that arose on account of the infusion of molecular biology into plant-breeding science constitute the new core of crop-genetic improvement, as an indispensable input for further research, side by side with both improved and exotic crop varieties that represent the operational background of crop-related research. Indeed, tools such as molecular markers, high-density genetic maps and structured mapping of populations provide breeders with the ability to "simultaneously define gene action and breeding value at hundreds of loci distributed relatively uniformly across entire genomes" (Moose and Munn 2008). The position of such research tools, as the groundwork of the innovation process in modern agricultural biotechnology, whether applied to conventional plant breeding or to transgenics, elevates the conditions surrounding their appropriation and further use to an essential issue.

Like the pressures and research needs which propelled the creation of the SNP Consortium with regard to the Human Genome, crop improvement research has also relied on partially-open institutions for the development of determinant molecular research tools. Rice research provides a useful example, demonstrating that, while patents lead to gains in terms of research efficiency and time, the existence of patent protection for the direct products of research is not a precondition for private sector involvement in innovation, while partially open information may also represent a prerequisite for further research and innovation within the world of high-sunk-cost bound specialised biotechnology research. Sequencing efforts are remarkable since the direct raw output of research activities is not subject to patent protection, while their potential impact on further research remains immense, especially with respect to the rice genome, viewed as the "Rosetta stone of cereals". Indeed, rice allows greater insight into the genetics of grasses and all major cereals, such as maize, barley or wheat; which are sizable commercial markets compared to the seemingly less lucrative rice market itself (Normile and Pennisi 2002). Map-based sequence information improves our knowledge of the location of all the genes in a genome, thereby extending the usefulness of molecular-marking technology, gaining in both accuracy and efficiency (Sasaki and Burr 2000), while also providing greatly improved estimates for gene-action-controlling traits of interest (Moose and Munn 2008). It thus constitutes an essential instrument for preliminary mandatory research in molecular plant breeding and transgenics. However, without further research aimed at the isolation and better understanding of a simply mapped pair of gene sequences, as well as strong arguments as to their precise utility and their detailed linkage to important crop traits, monopoly rights would typically not be granted over such a product (Pray and Naseem 2005).

The absence of intellectual property rights at the immediate end of the research and development chain did not preclude private investment in the map sequencing of the rice genome. Even though patents could theoretically have protected the entire array of tools that the rice-genome mapping research relied upon, a number of them remained in practice unpatented. The publicly available nature of such research tools has therefore also be seen as an important driver for innovation in terms of upstream molecular-biology research. Indeed, both the unpatented sequencing technologies developed by Frederick SANGER and Walter GILBERT, and the early public molecular markers, such as RFLPs (restriction fragment length polymorphisms), remain central to "automated genomic research" (Pray and Naseem 2005). The particularly interesting research collaboration that is the International Rice Genome Sequencing project (as well as other international attempts at sequencing) demonstrates that socially useful innovation may not only be achieved and sufficiently incentivised through partially-open information systems, but that it actually also heavily relies on such partial open mechanisms, since all parties recognise the role played by unpatented sequencing technology and access to other research teams' provisional research results in achieving the final objective of the international project.

However, the potential disregard or overlook of adequate rules of diffusion below the fence of raw research data still ought to be carefully considered. Within the framework of hybrid upstream research streams, while the data produced by consortiums and some selected research tools remains within the public domain, research efforts that aim to single out the exact utility of molecular biology research tools may lead to patent protection over what unvaryingly consists of platform technologies, to which a solid door of closed access shall be maintained. Within these hostile innovation environments, anti-commons emerge, characterised by the under-use and thus the under-production of innovative technologies, pushing legal scholarship to deplore the inappropriateness of such a restrictive approach to proprietary exclusiveness (Heller and Eisenberg 1998). Notably, the patent estate relating to the engineering and use of zinc-finger proteins, a technology that enables scientists to bind virtually any DNA sequence of interest, was initially owned by several different companies and academic institutions, a situation that raised concerns as to the prohibitive costs faced by subsequent users and developers in the negotiation of multiple licenses. This example is a classic scenario of the so-called “patent anti-commons” (Chandrasekharan et al. 2009). Another example is the controversy that surrounds research concerned with crop-variety genome mapping, much like the widely-cited example of the Human Genome and the uproar caused by the "Craig Venter" intellectual property protection strategy pattern in the mid 1990’s. Overall, the high number of patents on upstream molecular research tools, their concentration in the hands of small groups of enterprises, their alleged broad scope, the restrictive licensing practices (which drive away the innovation's availability), as well as the fierce enforcement strategies characterising protection, may indeed be cited as a cumulative rationale for assertions regarding the hindrance of socially desirable research and development.

#### Models responding to the partial paradigm breakdown for molecular breeding tools

Several options remain at hand to respond to the partial paradigm breakdown of plant breeding activities relying on molecular research tools, which yearn for a slice of partially open innovation structure. Alongside *ad hoc* institutional collaborations retaining a hybrid and semi-open nature, as epitomised by the aforementioned Rice Genome Sequencing project, regulatory provisos allowing for the development of the increasingly crucial molecular research tools can be found within the IP paradigm, more specifically in the recognition of a broad research exception in patent protection. The design of a specific regime for platform technologies can also be foreseen, ensuring their diffusion either through extensive yet well-defined statutory-use conditions, or through licensing protocols, intervening directly at the level of innovation diffusion. Indeed, an internationally recognised wide-reaching academic research exemption for biological research tools might not, according to certain commentators, properly discourage universities' institutional administrators from pursuing strong exclusive rights and licensing strategies (Lei *et al.*^2009^).

Legal solutions have in this context been primarily based upon liability regimes, moderating the risks of excluding third parties from accessing the technologies. Exemptions surrounding the grant of proprietary rights, namely for the purposes of research and experimental use, indeed not only acknowledge the derivate nature of conventional and molecular plant breeding activities, but also emphasise the reliance, production and conservation of agricultural biodiversity entailed within such particular research. In parallel to the official recognition of the breeders' exceptions within the international legal ethos, liability rules related to experimental uses in patent protection are recognised in the TRIPS Agreements' Article 30, whereby “*Members may provide limited exceptions to the exclusive rights conferred by a patent, provided that such exceptions do not unreasonably conflict with a normal exploitation of the patent and do not unreasonably prejudice the legitimate interests of the patent owner, taking account of the legitimate interests of third parties*.” This quasi-universal principle and its limitations leave non-negligible details to national or regional regulatory or jurisprudential fine-tuning. Indeed, even though both developed and developing countries alike have included an exception to patent protection for experimental use, based on the premise that the prevention of such use would frustrate the purpose of patent disclosure (Misati and Adachi 2010), this trend, and especially its reach, is nowhere universal.

The contours of the exception indeed show quite substantial differences in national legal orders, pertaining first to the question of the acts for which authorisation might not be sought (covering either research on, or extending up to research with, the innovation), while taking the researcher's purpose into account. For instance, a large number of countries, including the United States, operate a clear distinction between non-commercial research activities, which fall under the exception, and commercial endeavours, which require authorisation from the patent owner. However, delineating commercial and non-commercial research has proven to be a tricky and sensitive issue, opening jurisprudential debates on the merits of infringement claims (Misati and Adachi 2010). In the EU, common regional standards include Article 27 (b) Community Patent Convention 1975 as reaffirmed in 1989, as the common European statutory roots to the research exception, stating that "*the right conferred by a Community Patent does not extend to acts done for experimental purposes relating to subject matter of the patented invention*", but are also supplemented by national specificities and show quite the range of discrepancies. All EU Members, except Austria, indeed followed suit by using the exact wording of the Convention, while the Netherlands adopted somewhat narrower wording, and Belgium took the road of a broad research exception, covering both acts done with and on the protected innovation (Van Overwalle 2006).

In absence of legal constraints and/or in presence of a too narrow statutory exception for research undertaken with protected molecular research tools, an alternative solution might reside within the encouragement of formal agreements between institutions based upon partially-open innovation systems, discouraging the patenting of upstream research tools while fostering their common development and use. With regard to negotiated uses on the other hand, the navigation of licensing practices should be facilitated and universalised through pro-rata protocols including provisions against royalty stacking or even prefabricated licensing provisions encompassing *ex post* compensatory liability rules. In this respect the potential lying within an automatic, royalty-free license for research purposes in cases where the protected research tool is not made available on the market through a product or tool kit can be explored (Barton and Berger 2001), drawing perhaps from experiences with compulsory licensing mechanisms. The adjustment of existing rights and obligations between technology developers, holders and users, and the necessity to foster innovation while maintaining access to information and scientific progress, calls for a swift and equitable tailoring of license terms. The molecular plant-breeding innovation chain retains certain particularities that are difficult to capture within traditional IP tools, since there are often multiple types of protection surrounding a single product, whether at the actual physical level of possession of patented genetic-construct components such as promoters, or at the informational level of trademarked breeding methods or molecular markers.

## Conclusion

This paper has explored the effectiveness of the strong intellectual property paradigm to create incentives for innovation in the field of food and agricultural research, relying on massive inputs of plant-genetic resources into the research and development cycle. The main lesson is that there is not a single regime that fits all contexts best at the same time. The strong paradigm has proven very effective in the context of genetic engineering, but faces a systematic paradigm breakdown when it extends its regulatory scope over traditional mass selection operating for example in exchange networks of farmers’ landraces, where innovation often has a more collective, community-related nature. In these cases, a different form of intellectual-property protection, based on partially-open innovation systems, has proven more effective, even though its very existence is threatened by the global prevalence of the strong intellectual property paradigm, pushed into forced illegality and faced with significant disregard for the products of the innovation process. Genetic engineering and mass selection with naturally occurring landraces present two extreme cases.

Perhaps the most important part of modern agricultural research is happening between the two extremes of simple observation and deep molecular introspection. Conventional breeding, characterised by controlled hybridisation, and based upon the methodically-controlled crossing of specific varieties with other plant-genetic material, implies the repeated input of vast amounts of plant-genetic material, both from the pool of already domesticated varieties and from wild-plant genetic resources. Similarly, the use of molecular-biology-based gene marker technologies or bio-informatics to empower conventional breeding techniques is built around a process of cumulative incremental innovation, where the outputs of the research are used in turn as the direct inputs for the next innovation cycle. For these intermediary categories of plant improvement systems, both overly strong intellectual property rights and the absence of well delineated intellectual-property protection fail to provide incentives for investing in follow-on innovations. In particular, in cases where access to plant-genetic resources is a basic requirement to each new product development cycle, overly strong intellectual property rights might hamper or slow down the innovation process due to increased transaction costs generated by the need for complex licensing schemes for the use of these resources. Further, due to the interdependence of developed and developing countries for access to genetic resources for food and agriculture – for example situated in biodiversity hotspots in the South – special attention is required to create investment in genetic resources by countries that are situated far from the innovation frontier and which are less well placed to obtain and enforce strong intellectual property rights. Intellectual property rights legislation ought in this regard be recalibrated so as to incorporate farmers’ needs and rights, all the while constructing a well-suited reward regime to maintain adequate levels of genetic diversity that are not only important for farmers’ livelihoods or for environmental conservation policies, but also for the future of agricultural research and development efforts worldwide. As can be seen from the analysis in this article, neither strong intellectual property rights, nor their complete absence have proven an optimal fit from a legal and economic point view for the two intermediary categories of cumulative incremental innovation in agriculture research.

Finally, this conclusion leads us to recognise the role played by other arguments, beyond the legal analysis of optimal fit, when making regulatory decisions for intermediary categories of cumulative incremental innovation. Broader collective decisions on investments in the organisational and institutional infrastructures in scientific research will play a role in the choice of the most adequate legal framework for agrobiodiversity-based innovation in a given society. For instance, broader societal choices to be made for tackling the challenges posed by climate change for world-food security, may lead to decisions to increase investment in transgenic technologies developed either by private sector entities or by the public sector if granted sufficient funds to do so. It could also lead to increased investment in global collaboration between both public and private research institutions for experimental breeding, as a way to tackle these challenges. This paper does not advocate one or the other of these positions, but it does point to the fact that the choice of the best regulatory regime for agricultural biodiversity based innovation in a given society will depend on such broader debate, beyond the technical analysis of the legal and economic rationale alone.
